# HIV-1 virologic failure and acquired drug resistance among first-line antiretroviral experienced adults at a rural HIV clinic in coastal Kenya: a cross-sectional study

**DOI:** 10.1186/1742-6405-11-9

**Published:** 2014-01-23

**Authors:** Amin S Hassan, Helen M Nabwera, Shalton M Mwaringa, Clare A Obonyo, Eduard J Sanders, Tobias F Rinke de Wit, Patricia A Cane, James A Berkley

**Affiliations:** 1 KEMRI/Wellcome Trust Research Programme, Kilifi, Kenya; 2 Kilifi District Hospital, Kilifi, Kenya; 3 Centre for Clinical Vaccinology & Tropical Medicine, University of Oxford, Oxford, UK; 4 PharmAccess Foundation, Amsterdam, Netherlands; 5 Academic Medical Center, University of Amsterdam, Amsterdam, Netherlands; 6 Health Protection Agency, London, UK

**Keywords:** HIV, Virologic failure, Acquired drug resistance, Correlates, Rural, Kenya

## Abstract

**Background:**

An increasing number of people on antiretroviral therapy (ART) in sub-Saharan Africa has led to declines in HIV related morbidity and mortality. However, virologic failure (VF) and acquired drug resistance (ADR) may negatively affect these gains. This study describes the prevalence and correlates of HIV-1 VF and ADR among first-line ART experienced adults at a rural HIV clinic in Coastal Kenya.

**Methods:**

HIV-infected adults on first-line ART for ≥6 months were cross-sectionally recruited between November 2008 and March 2011. The primary outcome was VF, defined as a one-off plasma viral load of ≥400 copies/ml. The secondary outcome was ADR, defined as the presence of resistance associated mutations. Logistic regression and Fishers exact test were used to describe correlates of VF and ADR respectively.

**Results:**

Of the 232 eligible participants on ART over a median duration of 13.9 months, 57 (24.6% [95% CI: 19.2 – 30.6]) had VF. Fifty-five viraemic samples were successfully amplified and sequenced. Of these, 29 (52.7% [95% CI: 38.8 – 66.3]) had at least one ADR, with 25 samples having dual-class resistance mutations. The most prevalent ADR mutations were the M184V (n = 24), K103N/S (n = 14) and Y181C/Y/I/V (n = 8). Twenty-six of the 55 successfully amplified viraemic samples (47.3%) did not have any detectable resistance mutation. Younger age (15–34 vs. ≥35 years: adjusted odd ratios [95% CI], p-value: 0.3 [0.1–0.6], p = 0.002) and unsatisfactory adherence (<95% vs. ≥95%: 3.0 [1.5–6.5], p = 0.003) were strong correlates of VF. Younger age, unsatisfactory adherence and high viral load were also strong correlates of ADR.

**Conclusions:**

High levels of VF and ADR were observed in younger patients and those with unsatisfactory adherence. Youth-friendly ART initiatives and strengthened adherence support should be prioritized in this Coastal Kenyan setting. To prevent unnecessary/premature switches, targeted HIV drug resistance testing for patients with confirmed VF should be considered.

## Background

By the end of 2011, approximately 34 million people were living with HIV globally, with almost all (97%) coming from low and middle income countries (LMIC) [1]. In the same year, more than 8 million HIV-infected individuals in LMIC were receiving antiretroviral therapy (ART), up from just 400,000 in 2003 [2]. In Kenya, approximately 10,000 HIV-infected individuals were on ART in 2003. By the end of 2011, more than 400,000 individuals had initiated ART in the country [3]. The increase in the number of people with access to ART has resulted in substantial declines in HIV related incidence, morbidity and mortality [4-6]. However, emerging HIV-drug resistance and subsequent treatment failure threatens to reverse these gains. This is especially important in sub-Saharan Africa (sSA) where the scale up of ART has not always been done in tandem with the relevant support for virological monitoring and HIVDR testing.

Regular virological monitoring has been shown to be useful both in resource rich and resource limited settings [7,8]. However, due to cost implications, this is not currently recommended for routine use in most developing country settings. Instead, the World Health Organization (WHO) recommend use of clinical and immunological criteria to monitor treatment failure in resource limited settings [9]. These criteria have been demonstrated to be poor indicators of treatment failure, leading to missed opportunities or unnecessary medication switches [10-14], which not only increase treatment costs, but also limit future treatment options.

A systematic review of virological efficacy and drug resistance outcomes of patients on ART programmes in sSA has reported 76% virological suppression after 12 months on ART and 67% after 24 months [15]. Similarly, a recent systematic review from resource limited settings report HIV drug resistance of 11% in patients on ART for 12–23 months, 15% at 24–36 months and 21% at >36 months [16]. The most common resistance profiles identified include the M184V mutation (associated with nucleoside reverse transcriptase inhibitors; NRTIs), followed by the K103N mutation (associated with non-nucleoside reverse transcriptase inhibitors; NNRTIs). Thymidine analogue mutations (TAMs) and the K65R mutation were less common.

Emerging drug resistance and subsequent treatment failure poses a major concern for HIV programs in resource-limited settings where treatment options are limited. This study aimed to describe the prevalence and correlates of HIV-1 virologic failure and acquired drug resistance among first-line ART-experienced adults from a rural HIV clinic in coastal Kenya.

## Methods

### Study site

The study was carried out at the HIV clinic in Kilifi District Hospital; a rural public health facility located in Coastal Kenya. HIV services in the clinic are provided according to the Kenyan national guidelines [17]. In brief, immunological monitoring is recommended at enrolment into care and six-monthly (or when clinically indicated) thereafter. Individuals meeting the ART eligibility criteria (WHO clinical staging III/IV regardless of CD4 T-cell count or CD4 T-cell count of <350 cells/mm3 regardless of clinical staging) undergo ART preparedness counseling and are initiated on a standard first-line regimen.

At the time of the study, the national recommended first-line therapy comprised two NRTIs (stavudine/zidovudine and lamivudine) and one NNRTI (nevirapine/efavirenz). A gradual phase-out of stavudine as a first-line agent was recommended in mid-2010. Adherence counseling was done by nurse counselors. Individuals failing first-line therapy were switched to an alternative combination of two NRTIs and a boosted protease inhibitor (bPI) as the recommended second line of choice.

At the time of the study, routine HIV-1 virologic monitoring and drug resistance testing were not recommended in the Kenyan national guidelines. Targeted viral load monitoring was introduced in 2011. A switch to the recommended second line regimen was recommended for individuals with virologic failure (persistent viral load ≥1000 copies/ml).

For this study, remnant blood from routine CD4 count samples was centrifuged to obtain plasma, which was archived at −80 degrees centigrade and used for viral load quantification and HIVDR testing.

### Study design

An analytical cross-sectional study design was used. We included HIV-infected adults (≥15 years old) who had been on first-line ART for more than six months. Participants with a previous history of ART exposure for prevention of mother to child transmission (PMTCT) or for post-exposure prophylaxis (PEP), and those on second line regimens were excluded from the study.

Eligible participants were recruited in two phases. In the first cross-section, all consenting eligible participants were recruited between November 2008 and January 2009. At the same time, a prospective cohort was established in order to describe long-term outcomes of new clients enrolling for HIV care. All available plasma samples from participants recruited in the prospective cohort and meeting our eligibility criteria as at March 2011 were cross-sectionally retrieved.

### Sources of data

These have been previously described elsewhere [18]. In brief, socio-demographic data including date of birth, gender, marital status, level of education, religion and sub-location of residence were routinely collected using standardized questionnaires from all individuals at enrolment into HIV care by trained fieldworkers and counselors. Actual distance to the clinic was estimated from centroid co-ordinates of sub-locations in which participants resided to the clinic using ArcInfo (ArcCatalog version 9.2, ESRI Corp).

Clinical data including anthropometry, opportunistic infections, WHO staging, ART regimen, drug substitutions, drug pick up dates and appointments were routinely collected by trained clinicians on standardized forms at every clinic encounter. Hematology and CD4 T-cell count data were also collected. A trained data entry clerk entered these data into an electronic data system.

Medicine Possession Ratio (MPR), defined as the amount of time a participant is in possession of antiretrovirals divided by the time between ARV drug pick-ups, is increasingly being used as a proxy for assessing adherence in retrospective analyses. We therefore retrospectively retrieved pharmacy drug refill data from 12 months (or from the date of ART initiation if follow up period <12 months) prior to the date of sampling for every individual participant. MPRs were calculated as proportions of the total number of days between drug pick-ups less the equivalent number of days in possession of ART divided by the time between drug pick-ups for all visits. A mean MPR for each individual was computed, subtracted from 100% and stratified to satisfactory (≥95%) and unsatisfactory (<95%) adherence according to previously published conventions [19,20].

### Outcome definitions

The primary outcome was HIV-1 virologic failure (VF), defined as a one-off HIV-1 plasma RNA viral load of ≥400 copies/ml. Viral load quantification was done using an in-house assay. In brief, a multiplex real time quantitative probe-based assay with an internal control and a series of quantified HIV-1 standards was used to determine virus concentration. The assay is designed to quantify HIV-1 plasma RNA for thresholds of 100 – 10,000,000 copies/ml.

The secondary outcome was acquired drug resistance (ADR), determined by HIV-1 genotyping. Genotypic resistance testing was done for all samples with VF using an in-house assay which has been described elsewhere [21]. In brief, the assay amplifies and sequences part of the *pol* sub-genomic region containing the protease and part of the reverse transcriptase genes. Sequences were manually edited and assembled against a reference sequence using Sequencher software (GeneCodes, version 4.1). Sequences were submitted to the Stanford HIV drug resistance database to identify and interpret HIV-1 drug resistance mutations [22,23].

Viral subtypes were identified by ‘Subtype Classification Using Evolutionary Algorithm tool (SCUEAL)’ (http://www.datamonkey.org/dataupload.php) [24].

### Sample size

A *post-hoc* sample size calculation was done to describe whether the data would produce results with sufficient statistical precision. This study assessed for HIV-1 VF among 232 patients on first-line antiretroviral therapy. Assuming a HIV-1 VF prevalence of 24% after a median follow-up period of 12 months on ART in our setting (based on 76% virological suppression after 12 months on ART as reported in a systematic review elsewhere [15]), the risk of 232 ART naïve adults started on first-line regimen and developing VF over a median follow up period of twelve months could be estimated with a precision of +/−6% at 95% confidence level.

### Data analysis

Continuous data are presented using medians (inter-quartile ranges, IQR). Because of the relatively small sample size, and except for marital status, all the exposure variables were grouped into two categories. Continuous data were stratified into two categories, using the median as the guide to the stratification threshold. Frequencies and column percentages were used to describe categorical data.

The prevalence of HIV-1 VF was determined as a percentage of plasma samples with detectable viral load ≥400 copies/ml. The prevalence of ADR was determined as a percentage of samples with detectable resistance associated mutations (as identified by the Stanford HIV drug resistance database) over the total number of samples with VF that were successfully amplified and sequenced.

Univariable and multivariable logistic regression was used to determine correlates of VF. Correlates with a likelihood ratio test (LRT) p-value of <0.05 from the univariable analysis were carried to the multivariable models using the forward stepwise approach. Crude and adjusted odd ratios (OR), 95% confidence intervals (CI) and LRT p-values were presented. Because of the low frequency observed, the Fishers exact test was used to assess for correlates of ADR among all the participants included in the study. Frequencies, row percentages and the Fisher’s exact p-values were presented.

From a public health perspective, the Kenyan national ART guidelines recommend use of persistent viral loads of ≥1000 copies/ml as indicative of VF [25]. For comparison purposes with the study outcomes, this definition was also considered in the analyses, albeit from a one-off sampling approach.

All analyses were carried out using STATA statistical software (STATA Intercooled version 11, StataCorp, College Station, Texas, USA).

### Ethical considerations

The Kenya Medical Research Institute (KEMRI) Scientific Steering Committee and the National ethics review committee provided scientific and ethics approval respectively (SSC No. 1341). All the participants provided written informed consent.

## Results

### Study population characteristics

Overall, 232 adults on first-line ART for a median duration of 13.9 (IQR: 10.0 – 18.3) months were recruited. The characteristics of participants recruited in the first cross-section were not substantially different from those recruited in the second cross-section (Table 1).

**Table 1:** Distribution of characteristics among first-line antiretroviral experienced adults on care at a rural HIV clinic in coastal Kenya (N = 232)

**Characteristic**	**Categories**	**Frequency [column %]**		
		**Cross-section 1 (n = 86)**	**Cross-section 2 (n = 146)**	**Total (n = 232)**
**Gender**	Male	16 [18.6]	38 [26.0]	54 [23.3]
	Female	70 [81.4]	108 [74.0]	178 [76.7]
*Age (years)	Median	36.5	39.3	38.5
	[IQR]	[31.4 – 44.4]	[32.7 – 46.1]	[32.2 – 44.8]
**Age group (years)**	15.0 – 34.9	33 [38.4]	47 [32.2]	80 [34.5]
	≥ 35.0	53 [61.6]	99 [67.8]	152 [65.5]
**Marital status**	Single	10 [11.6]	9 [6.2]	19 [8.2]
	Married (monogamous/polygamous)	52 [60.5]	80 [54.8]	132 [56.9]
	Separated/divorced/widowed	24 [27.9]	57 [39.0]	81 [34.9]
**Religion**	Christian	64 [74.4]	88 [60.3]	152 [65.5]
	Muslim	13 [15.1]	28 [19.2]	41 [17.7]
	Others	9 [10.5]	30 [20.6]	39 [16.8]
**Education status**	Primary schooling/less	68 [79.1]	119 [81.5]	187 [80.6]
	Secondary/higher	18 [20.9]	27 [18.5]	45 [19.4]
*Distance (km)	Median	7.8	7.8	7.8
	[IQR]	[2.2 – 21.0]	[2.2 – 13.4]	[2.2 – 15.7]
**Group distance (km)**	< 10.0	50 [58.1]	98 [67.1]	148 [63.8]
	≥ 10.0	36 [41.9]	48 [32.9]	84 [36.2]
**Starting 1**^**st **^**line regimen**	Zidovudine based	37 [43.0]	81 [55.5]	118 [50.9]
	Stavudine based	49 [57.0]	65 [44.5]	114 [49.1]
*Baseline WHO staging	I/II	41 [47.7]	90 [61.6]	131 [56.5]
	III/IV	44 [51.2]	56 [38.4]	100 [43.1]
	Missing	1 [0.0]	0 [0.0]	1 [0.4]
*Baseline BMI (Kg/m^**2**^**)**	Median	19.3	19.0	19.3
	(IQR)	[17.6 – 20.7]	[17.3 – 21.1]	[17.4 – 21.1]
**Baseline BMI groups (Kg/m**^**2**^**)**	< 18.5	32 [37.2]	63 [43.2]	95 [41.0]
	≥ 18.5	53 [61.6]	83 [56.9]	136 [58.6]
	Missing	1 [1.2]	0 [0.0]	1 [0.4]
*Baseline CD4 count (cells/uL)	Median	124	126	124
	(IQR)	[61 – 197]	[35–193]	[40–196]
**Baseline CD4 groups (cells/uL)**	< 100	33 [38.4]	63 [43.2]	96 [41.4]
	≥ 100	51 [59.3]	83 [56.9]	134 [57.8]
	Missing	2 [2.3]	0 [0.0]	2 [0.9]
*Duration on ART (months)	Median	13.3	15.0	13.9
	[IQR]	[9.0 – 16.1]	[10.8 – 20.3]	[10.0 – 18.3]
**Group duration on ART (months)**	<12.0	32 [37.2]	49 [33.6]	81 [34.9]
	≥ 12.0	54 [62.8]	97 [66.4]	151 [65.1]
**Drug substitution**	No	68 [79.1]	100 [68.5]	168 [72.4]
	Yes	18 [20.9]	46 [31.5]	64 [27.6]
**WHO staging**	Stage I/II	54 [62.8]	78 [53.4]	132 [56.9]
	Stage III/IV	32 [37.2]	68 [46.6]	100 [43.1]
*BMI (Kg/m^**2**^**)**	Median	21.2	21.1	21.1
	(IQR)	[19.2 – 22.2]	[19.4 – 24.5]	[19.4 – 23.6]
**BMI groups (Kg/m**^**2**^**)**	< 18.5	12 [14.0]	24 [16.4]	36 [15.5]
	≥ 18.5	74 [86.1]	122 [83.6]	196 [84.5]
*CD4 count (cells/uL)	Median	282	288	286
	(IQR)	[205–419]	[193–387]	[199–388]
**CD4 groups (cells/uL)**	< 350.0	49 [57.0]	82 [56.2]	131 [56.5]
	≥ 350.1	29 [33.7]	41 [28.1]	70 [30.2]
	Missing	8 [9.3]	23 [15.8]	31 [13.4]
**MPR adherence**	≥ 95% (Satisfactory)	59 [68.6]	122 [83.6]	181 [78.0]
	< 95% (Unsatisfactory)	24 [27.9]	19 [13.0]	43 [18.5]
	Missing	3 [3.5]	5 [3.4]	8 [3.5]

*Baseline* refers to indicators at ART initiation; *Follow up* refers to indicators at the time of sampling; *Median [IQR, Inter-quartile ranges] for continuous variables; BMI (Body Mass Index); WHO (World Health Organization); MPR (Medicine Possession Ratio); Zidovudine based (plus lamivudine/Nevirapine [n = 107] or lamivudine/efavirenz [n = 11]); stavudine based (plus lamivudine/Nevirapine [n = 111] or lamivudine/efavirenz [n = 3]).

The majority of the participants were female (n = 178 [77%]), aged more than 35 years (n = 152 [66%]), married (n = 132 [57%]), with a primary education or less (n = 187 [81%]) and living within 10 kilometers of the clinic (n = 148 [64%]) (Table 1). Half of the participants (n = 118) initiated ART on a zidovudine-based regimen (plus lamivudine/nevirapine, n = 107 [46%] or lamivudine/efavirenz, n = 11 [5%]); the other half (n = 114) started on a stavudine-based regimen (plus lamivudine/nevirapine, n = 111 [48%] or lamivudine/efavirenz, n = 3 [1%]). Over the follow up duration on ART, 64 (28%) participants had undergone at least one drug substitution and 43 (19%) had an average unsatisfactory MPR adherence.

### *HIV-1* virologic failure

Of the 232 samples that underwent HIV-1 RNA viral load quantification, 57 (24.6% [95% CI: 19.2 – 30.6]) demonstrated VF. In univariable analysis, MPR adherence, age group and marital status were significantly correlated with VF. In multivariable analysis, only MPR adherence and age group remained independently associated with VF (Table 2). Participants with an average unsatisfactory MPR adherence had three-fold odds of having VF, compared to those with an average satisfactory MPR adherence (aOR [95% CI], p-value: 3.0 [1.5 – 6.5], p = 0.003). Likewise, participants aged ≥35 years had 70% lower odds of having VF, compared to those aged 15 – 34 years old (0.3 [0.1 – 0.6], p < 0.001). Adjusting for age attenuated the effect of marital status on VF towards the null (Separated/divorced/widowed vs. single, 0.4 [0.1 – 1.2], p = 0.137).

**Table 2:** Logistic regression analysis describing correlates of HIV-1 virologic failure (viral load ≥400 copies/ml) among first-line antiretroviral experienced adults at a rural HIV clinic in coastal Kenya (N = 232)

			**Logistic univariable analysis**			**Logistic multivariable analysis (n = 201)**		
**Risk factors**	**Categories**	**Viraemia, n = 57 [%]**	**Crude OR**	**95% C.I**	*P-value	**Adjusted OR**	**95% C. I**	*P-value
**Gender**	Male	12/54 [22.2]	1.0					
	Female	45/177 [25.3]	1.2	0.6 – 2.4	0.645	-	-	-
**Age group (years)**	15.0 – 34.9	31/80 [38.8]	1.0			1.0		
	≥ 35.0	26/152 [17.1]	0.3	0.2 – 0.6	<0.001	0.3	0.2 – 0.7	0.002
**Marital status**	Single	8/19 [42.1]	1.0					
	Married (monogamous/polygamous)	36/132 [27.3]	0.5	0.2 – 1.4				
	Separated/Divorced/Widowed	13/81 [16.1]	0.3	0.1 – 0.8	0.034	-	-	-
**Religion**	Christian	38/152 [25.0]	1.0					
	Muslim	7/41 [17.1]	0.6	0.3 – 1.5				
	Others	12/39 [30.8]	1.3	0.6 – 2.9	0.345	-	-	-
**Education status**	Primary schooling/Less	46/187 [24.6]	1.0					
	Secondary/Higher	11/45 [24.4]	1.0	0.5 – 2.1	0.983	-	-	-
**Group distance (km)**	<10.0	37/148 [25.0]	1.0					
	≥ 10.0	20/84 [23.8]	0.9	0.5 – 1.8	0.839	-	-	-
**Starting 1**^**st **^**line regimen**	Zidovudine based	33/118 [28.0]	1.0					
	Stavudine based	24/114 [21.1]	0.7	0.4 – 1.3	0.221	-	-	-
***Baseline *** **WHO staging**	I/II	28/131 [21.4]	1.0					
	III/IV	28/100 [28.0]	1. 4	0.8 – 2.6	0.246	-	-	-
***Baseline *** **BMI groups (Kg/m**^**2**^**)**	< 18.5	26/95 [27.4]	1.0					
	≥ 18.5	30/136 [22.1]	0.8	0.4 – 1.4	0.356	-	-	-
***Baseline *** **CD4 groups (cells/uL)**	< 100	25/96 [26.0]	1.0					
	≥ 100	30/134 [22.4]	0.8	0.4 – 1.5	0.523	-	-	-
**Duration on ART (months)**	<12.0	23/81 [28.4]	1.0					
	≥ 12.0	34/151 [22.5]	0.7	0.4 - 1.4	0.325	-	-	-
**Drug substitution**	No	40/168 [23.8]	1.0					
	Yes	17/64 [26.6]	1.2	0.6 – 2.2	0.665	-	-	-
***Follow up *** **WHO staging**	Stage I/II	29/132 [22.0]	1.0					
	Stage III/IV	28/100 [28.0]	1.4	0.8 – 2.5	0.292	-	-	-
***Follow up *** **BMI groups (Kg/m**^**2**^**)**	< 18.5	10/36 [27.8]	1.0					
	≥ 18.5	47/196 [24.0]	0.8	0.4 – 1.8	0.627	-	-	-
***Follow up *** **CD4 groups (cells/uL)**	< 350	35/131 [26.7]	1.0					
	≥ 350	12/70 [17.1]	0.6	0.3 – 1.2	0.120	-	-	-
**MPR adherence**	≥ 95% (Satisfactory)	34/181 [18.8]	1.0			1.0		
	<95% (Unsatisfactory)	18/43 [41.9]	3.1	1.5 – 6.3	0.002	3.0	1.5 – 6.5	0.003

*Baseline* refers to indicators at ART initiation; *Follow up* refers to indicators at the time of sampling; *Likelihood Ratio Test p-value; BMI (Body Mass Index); WHO (World Health Organization); MPR (Medicine Possession Ratio). Missing data; Baseline WHO staging (n = 1 [0.4%]), Baseline BMI (n = 1 [0.4%]), Baseline CD4 count (n = 2 [0.9%]), CD4 count (n = 31 [13.4%]) and MPR adherence (n = 8 [3.5%]).

### HIV-1 acquired drug resistance

Fifty-five of the 57 samples with VF were successfully amplified and sequenced for HIV drug resistance testing. Of the 55 samples that were successfully amplified and sequenced, 29 (52.7% [95% CI: 38.8 – 66.3]) had at least one detectable HIV-1 resistance associated mutation, giving an overall ADR prevalence of 12.5% (95% CI: 8.5 – 17.5) among all participants included in the study. While all 29 samples had mutations conferring resistance to NNRTIs, 25 (86%) of those with resistance had dual-class (both NRTIs and NNRTIs) mutations (Table 3). The most prevalent variant were the M184V mutation (n = 24), the K103N/S mutation (n = 14) and the Y181C/Y/I/V mutation (n = 8) within the reverse transcriptase genome. Thymidine analogue mutations (TAMs) were present in 4 participants. Twenty-six of the 55 successfully amplified viraemic samples (47.3%) did not have any detectable resistance associated mutations.

**Table 3:** Distribution and characteristics of first-line antiretroviral experienced adults with HIV-1 acquired drug resistance mutations from a rural HIV clinic in coastal Kenya

**No.**	**Gender**	**Age (years)**	**Sample date**	**ART date**	**ART duration**^**#**^	**Viral load**	Subtype*	**NRTI mutations**	**NNRTI mutations**
**1.**	Male	30.5	28-Nov-08	28-Mar-08	8.0	697796	Complex	D67DG	K103N, G190A
**2.**	Female	38.5	02-Dec-08	22-May-08	6.4	5006	A1	M184V	K103N, K238T
**3.**	Male	36.6	06-Jan-09	11-Apr-08	8.9	861730	A1	M184V	Y188L
**4.**	Female	33.4	17-Nov-08	19-May-08	6.0	64435	A1	M184V, K219EK	V108IV, Y181CY, G190AG
**5.**	Female	44.5	16-Dec-08	26-Nov-07	12.7	3087	A1	M184V	G190A
**6.**	Female	26.5	17-Dec-08	01-Nov-07	13.5	1158	D	M184V	K103N, K238T
**7.**	Female	22.5	05-Dec-08	30-Oct-07	13.2	4051	A1	M184V	K103N
**8.**	Female	48.4	12-Nov-08	02-Jul-07	16.4	5274	A1	M184V	K103N, M230LM
**9.**	Female	24.4	12-Nov-08	29-Aug-07	14.5	24529	D	T69NT, M184V	K103N
**10.**	Female	63.4	21-Nov-08	03-May-07	18.7	576	A1, AE		K103N
**11.**	Female	25.6	14-Jan-09	22-Oct-07	14.8	219766	A-ancestral, A1	M184V, T215Y	Y181C
**12.**	Female	31.3	07-Oct-10	22-Aug-08	25.5	315800	A1	M184V	K103N
**13.**	Female	34.0	22-Jun-10	07-Oct-09	8.5	17248	A1	M184V	Y181C
**14.**	Female	25.2	13-Sep-10	19-Aug-09	12.8	109090	D	M184V	V106A, F227L
**15.**	Female	51.2	10-Aug-10	15-Jun-09	13.8	5396	A1	M184V	K103N
**16.**	Female	23.7	08-Mar-11	22-Sep-09	17.5	12408	A1	M184V	Y181C
**17.**	Female	32.1	06-Jul-10	15-Dec-08	18.7	199362	A1	M41L, D67N, K70R, M184V, T215Y, K219Q	Y181IV
**18.**	Female	31.8	23-Mar-11	27-Feb-09	24.8	834656	A-ancestral, A1		K101E, G190A
**19.**	Female	38.7	16-Feb-10	22-May-09	8.9	34186	A2	M184V	V106A
**20.**	Female	41.8	20-Apr-10	22-Jun-09	9.9	4729	A1	M184V	Y181C
**21.**	Female	31.7	04-Mar-11	24-Mar-09	23.3	20392	A1	M184V	V106A
**22.**	Female	15.1	16-Mar-10	13-Apr-09	11.1	1046760	A1	M184V	K103S, G190A
**23.**	Female	31.3	29-Sep-10	11-Jan-10	8.6	129493	A1, A2		K103N
**24.**	Female	41.7	11-Mar-11	18-Aug-09	18.7	1452	A, A1	M184V	Y181C
**25.**	Female	17.8	07-Mar-11	08-Dec-09	14.9	130222	A1	M184V	K103N, Y318FY
**26.**	Female	25.7	04-Mar-11	13-Apr-10	10.7	47795	A1	M184V	K101Q, G190A
**27.**	Female	21.7	11-Mar-11	01-Sep-09	18.3	2029880	A1	L74V, M184V	K103N, Y181C
**28.**	Female	48.1	02-Aug-10	08-Oct-08	21.8	72430	A1		G190A
**29.**	Male	29.5	14-Dec-10	06-Jan-10	11.2	74152	A, A1	K65R, M184V	K103N

^#^Time (in months) since patient started taking antiretroviral therapy, *HIV-1 subtypes identified using the ‘Subtype Classification Using Evolutionary ALgorithm (SCUEAL)’ tool, available at (http://www.datamonkey.org/dataupload_scueal.php).

Viral load, MPR adherence and age group were strongly associated with ADR (Table 4). Participants with higher viral loads (≥ 4.0 log copies/ml) had a higher prevalence of ADR, compared to those with lower viral loads (< 4.0 log copies/ml), (frequency [%]: 20 [62.5] vs 9 [4.6], p < 0.001). Likewise, participants with unsatisfactory MPR adherence had a higher prevalence of ADR, compared to those with satisfactory MPR adherence (frequency [%]: 12 [27.9] vs 17 [9.5], p = 0.004). Similarly, participants aged 15– 34 had a higher prevalence of ADR compared to those aged ≥35 years (frequency [%]: 19 [23.8] vs 10 [6.7], p < 0.001).

**Table 4:** Distribution and correlates of HIV-1 acquired drug resistance among first-line antiretroviral experienced adults at a rural HIV clinic in coastal Kenya (N = 230)

		**Acquired drug resistance [row %]**		
**Risk factors**	**Categories**	**No (n = 201)**	**Yes (n = 29)**	*P-value
**Gender**	Male	51 [94.4]	3 [5.6]	
	Female	150 [85.2]	26 [14.8]	0.100
**Age group (years)**	15.0 – 34.9	61 [76.3]	19 [23.8]	
	≥ 35.0	140 [93.3]	10 [6.7]	<0.001
**Marital status**	Single	13 [68.4]	6 [31.6	
	Married (monogamous/polygamous)	113 [86.3]	18 [13.7]	
	Separated/divorced/widowed	75 [93.8]	5 [6.3]	0.013
**Religion**	Christian	132 [87.4]	19 [12.6]	
	Muslim	36 [87.8]	5 [12.2]	
	Others	33 [86.8]	5 [13.2]	1.000
**Education status**	Primary schooling/less	162 [87.6]	23 [12.4]	
	Secondary/higher	39 [86.7]	6 [13.3]	0.807
**Group distance (km)**	<10.0	128 [86.5]	20 [13.5]	
	≥10.0	73 [89.0]	9 [11.0]	0.681
**Starting 1**^**st **^**line regimen**	Zidovudine based	102 [86.4]	16 [13.6]	
	Stavudine based	99 [88.4]	13 [11.6]	0.695
***Baseline *** **WHO staging**	I/II	117 [90.0]	13 [10.0]	
	III/IV	83 [83.8]	16 [16.2]	0.228
***Baseline *** **BMI groups (Kg/m**^**2**^**)**	< 18.5	82 [88.2]	11 [11.8]	
	≥ 18.5	118 [86.8]	18 [13.2]	0.841
***Baseline *** **CD4 groups (cells/uL)**	<100	82 [86.3]	13 [13.7]	
	> 100	118 [88.7]	15 [11.3]	0.683
**Duration on ART (months)**	< 12.0	70 [86.4]	11 [13.6]	
	≥ 12.0	131 [87.9]	18 [12.1]	0.836
**Drug substitution**	No	145 [87.4]	21 [12.7]	
	Yes	56 [87.5]	8 [12.5]	1.000
***Follow up *** **WHO staging**	Stage I/II	119 [91.5]	11 [8.5]	
	Stage III/IV	82 [82.0]	18 [18.0]	0.044
***Follow up *** **BMI groups (Kg/m**^**2**^**)**	< 18.5	30 [83.3]	6 [16.7]	
	≥ 18.5	171 [88.1]	23 [11.9]	0.417
***Follow up *** **CD4 groups (cells/uL)**	< 350	110 [84.6]	20 [15.4]	
	≥ 350	64 [91.4]	6 [8.6]	0.193
**MPR adherence**	≥ 95% (Satisfactory)	162 [90.5]	17 [9.5]	
	< 95% (Unsatisfactory)	31 [72.1]	12 [27.9]	0.004
**Viral load (log 10, copies/ml)**	0.00 – 4.00	189 [95.5]	9 [4.6]	
	> 4.00	12 [37.5]	20 [62.5]	<0.001

*Baseline* refers to indicators at ART initiation; *Follow up* refers to indicators at the time of sampling; *Fisher’s exact p-value; BMI (Body Mass Index); WHO (World Health Organization) MPR (Medicine Possession Ratio). Missing data; Baseline WHO staging (n = 1 [0.4%]), Baseline BMI (n = 1 [0.4%]), Baseline CD4 count (n = 2 [0.9%]), CD4 count (n = 31 [13.4%]) and MPR adherence (n = 8 [3.5%]).

**Figure 1:**
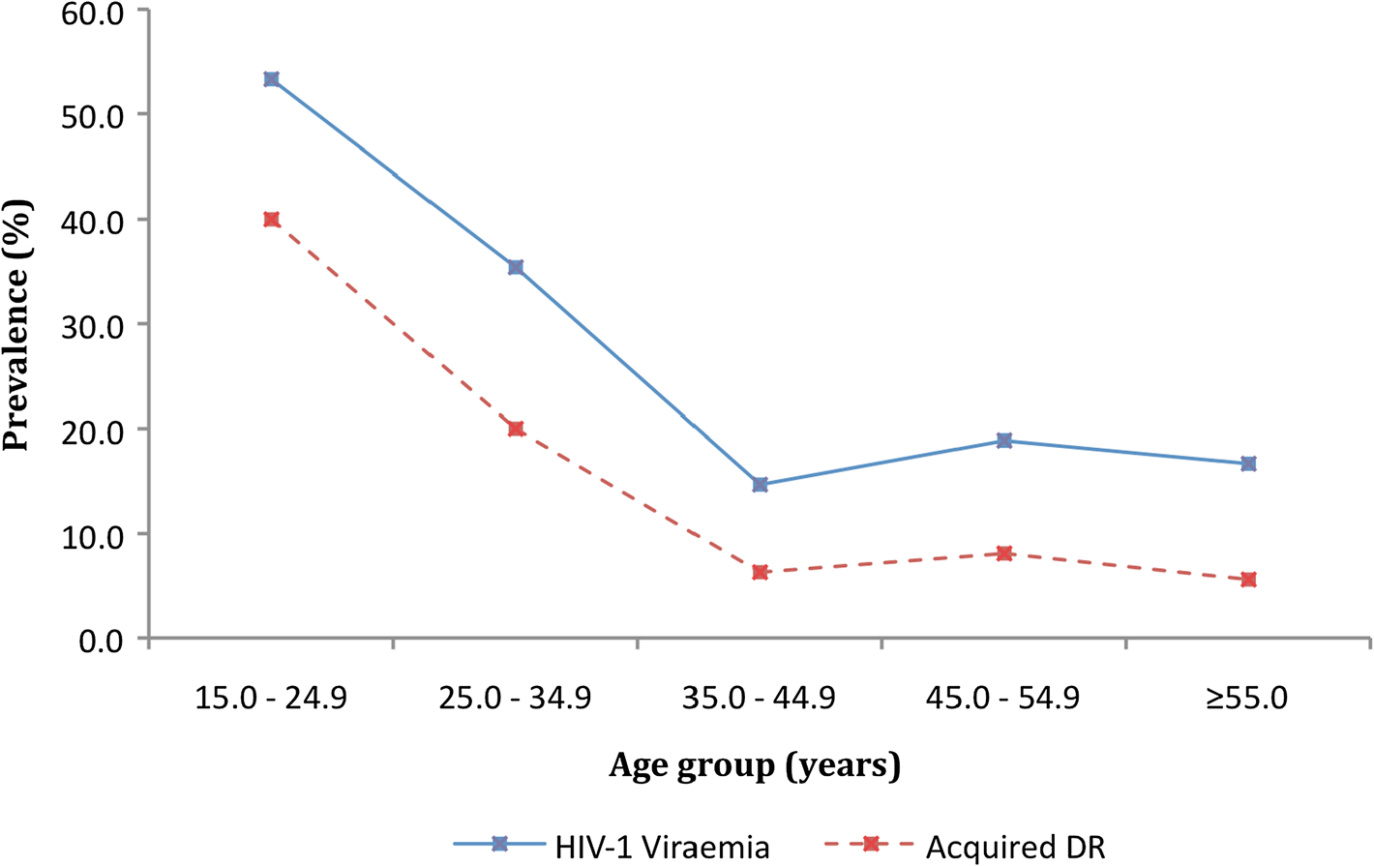
Overall prevalence and distribution of HIV-1 Viraemia (viral load ≥400 copies/ml) and acquired drug resistance by age group, among first-line antiretroviral experienced adults at a rural HIV clinic in coastal Kenya (N=232).

Age was further stratified to 10-year age bands and its association with VF and ADR explored. The overall prevalence of VF and ADR was highest in participants aged 15–24 years (53.3% and 40.0% respectively) (Figure 1). Strong evidence of a decreasing trend in prevalence of VF and ADR with increasing age groups (non-parametric test for trend, p = 0.004 and p < 0.001 respectively) was also observed.

For comparison purposes, the above analyses were repeated using viral load threshold of ≥1000 copies/ml to define VF. Of the 232 participants, 48 (20.7% [95% CI: 15.7 – 26.5]) met this criterion of HIV-1 VF. Of these, 28 (58.3% [95% CI: 43.2 – 72.4]) had at least one detectable HIV-1 resistance mutation. Correlates of VF (Additional file 1: Table S1) and ADR (Additional file 1: Table S2) remained the same as those observed when using this study’s outcome definition of ≥400 copies/ml for VF.

## Discussion

Findings from a HIV clinic in a rural district hospital in coastal Kenya suggest that one in every four adults on first-line antiretroviral regimen for an average of 14 months had VF, with half of those with VF harboring at least one HIV resistance-associated mutation. The most prevalent mutations observed confer high-level resistance to NRTIs (specifically lamivudine, in the case of the M184V mutation) and NNRTIs (specifically nevirapine and efavirenz, in the case of the K103N/S mutation and the Y181C/Y/I/V mutation). These results are consistent with findings from systematic reviews of studies on virological efficacy and drug resistance from other resource limited settings [15,26]. The non-complex resistance patterns observed could possibly indicate an advantage of the current recommended first-line regimen in this setting.

The WHO recommends use of drug-refill data as an early warning indicator (EWI) of HIV treatment failure and drug resistance [27]. Recent EWI analyses on prospective HIVDR data from 6 African countries suggest an advantage of MPR over on-time drug pick-up in identifying participants at risk for developing HIV drug resistance [28]. For this reason, MPR was used and indeed identified as a practical alternative parameter for assessing adherence, with strong correlation with both VF and ADR in this setting. Similar findings have also been reported elsewhere [19,29-31].

The current study also indicated younger age as a strong risk factor for both HIV-1 VF and ADR. In fact, half of all participants aged 15–24 years had VF, while two in every five had acquired at least one drug resistant strain. Data from a developed setting suggests that the youth face a complex myriad of challenges including peer-related stigma, disclosure, adherence, sexual, reproductive and gender health concerns [32]. If applicable, then these challenges may indirectly contribute to the high burden of virological treatment failure and ADR in our setting.

Higher viral load was strongly associated with ADR, which is consistent with literature [33]. Of interest however, is the finding that only half of participants with VF had detectable ADR. Indeed, if the Kenyan national recommendation of ≥1000 copies/ml was used to suggest VF in this study [25], and assuming sustained viral replication, then a fifth of the participants would have VF. Of these, only 58% had detectable ADR mutations. These data may therefore suggest that up to 42% of participants would have potentially been switched to the more expensive 2^nd^ line regimen prematurely or unnecessarily, thus exhausting and limiting treatment options. This is especially risky in this setting where the only currently recommended second line option is the bPIs, with costs prohibiting the range of other potential alternative regimens.

The findings of this study should be interpreted in light of several limitations. Firstly, the cross-sectional study design and the one-off sampling strategy warrant caution in the interpretation of our findings, as follow up samples were not available to confirm VF. Consequently, blips in viral load cannot be ruled out [34]. It is acknowledged that occasionally, viral blips can occur even during effective treatment [35,36]. This may have resulted to an overestimation of the true burden of VF in this population. In addition, focusing on participants with a median ART follow up duration of more than a year potentially excludes those who may have died or were lost to follow-up within a year of ART due to treatment failure. This may have resulted in an underestimation of the true burden of VF and ADR in this population.

Secondly, it may be argued that the participants may not have achieved virological suppression in the first place, even after being on ART for more than 6 months. This may possibly be attributed to the effect of persisting HIV-1 primary or transmitted resistance mutations, which have been reported to be on the increase in some parts of sSA [37,38]. Transmitted resistant strains have been shown to contribute to VF in clients on ART [39-41]. However, our concurrent data suggest low levels (<5%) of transmitted drug resistance in this rural coastal Kenyan population [42].

Lastly, stigma, disclosure, sexual orientation, reproductive health and gender issues are potential concerns that may contribute to the burden of VF and ADR, especially among the young adults in this setting. Data on these factors were not captured and hence not considered in the analyses.

## Conclusions

In conclusion, levels of HIV-1 VF and ADR observed from this rural HIV clinic in coastal Kenya were comparable to those observed in other resource-limited settings. High levels of VF and ADR were observed among younger patients and those with unsatisfactory adherence. Implementation of youth-friendly ART initiatives (e.g. social networks, support clubs) are therefore warranted in this setting. Strengthened adherence support should also be prioritized, more so in cases of suspected virologic treatment failure and before treatment switches. However, if virologic failure is confirmed, targeted HIVDR testing should be considered to prevent unnecessary/premature switches.

## Competing interests

The authors declare that they have no competing interest.

## Authors’ contributions

JAB and PAC conceived the study. ASH coordinated the data/sample collection, analyzed the data and prepared the draft manuscript. SM, HN and CAO assisted with the coordination of the data and sample collection. JAB, PAC, ES and TFRW provided guidance and mentorship during the implementation of the study. All authors reviewed and approved the final manuscript.

## Supplementary Material

Additional file 1: Table S1 Logistic regression analysis describing correlates of HIV-1 virologic failure (viral load ≥1000 copies/ml) among first line antiretroviral experienced adults at a rural HIV clinic in coastal Kenya (N=232). **Table S2.** Distribution and correlates of HIV-1 acquired drug resistance (if genotyping was only done for samples with viral load ≥1000 copies/ml) among first-line antiretroviral experienced adults at a rural HIV clinic in coastal Kenya (N=230). Click here for file

## Sequence data

GenBank accession numbers [GenBank: KC568476 – KC568530].
