# Supplementary material for: HIV-1 virologic failure and acquired drug resistance among first-line antiretroviral experienced adults at a rural HIV clinic in coastal Kenya: a cross-sectional study
Source: AIDS Res Ther. 2014 Jan 23;11:9. doi: 10.1186/1742-6405-11-9 (PMC3922732; doi:10.1186/1742-6405-11-9)
Supplement: Additional file 1: Table S1 — Logistic regression analysis describing correlates of HIV-1 virologic failure (viral load ≥1000 copies/ml) among first line antiretroviral experienced adults at a rural HIV clinic in coastal Kenya (N=232). Table S2. Distribution and correlates of HIV-1 acquired drug resistance (if genotyping was only done for samples with viral load ≥1000 copies/ml) among first-line antiretroviral experienced adults at a rural HIV clinic in coastal Kenya (N=230). [file 1742-6405-11-9-S1.pdf]

Supplementary table 1: Logistic regression analysis describing correlates of HIV-1 virologic failure (viral load ≥1000 copies/ml) among first line antiretroviral experienced adults at a rural HIV clinic in coastal Kenya (N=232).

			Logistic univariable analysis			Logistic multivariable analysis (n=201)		
**Characteristic**	**Categories**	**Viraemia, n=48 [%]**	**Crude OR**	**95% C.I**	***P-value**	**Adjusted OR**	**95% C. I**	***P-value**
**Gender**	Male	11/54 [20.4]	1.0					
	Female	37/178 [20.8]	1.0	0.5 – 2.2	0.947	-	-	-
**Age group (years)**	15.0 – 34.9	28/80 [35.0]	1.0			1.0		
	≥ 35.0	20/152 [13.2]	0.3	0.1 – 0.5	<0.001	0.2	0.1 – 0.5	<0.001
**Marital status**	Single	7/19 [36.8]	1.0					
	Married (monogamous/polygamous)	31/132 [23.5]	0.5	0.2 – 1.5				
	Separated/Divorced/Widowed	10/81 [12.4]	0.2	0.1 – 0.8	0.029	-	-	-
**Religion**	Christian	32/152 [21.1]	1.0					
	Muslim	6/41 [14.6]	0.6	0.2 – 1.7				
	Others	10/39 [25.6]	1.3	0.6 – 2.9	0.459	-	-	-
**Education status**	Primary schooling/Less	37/187 [19.8]	1.0					
	Secondary/Higher	11/45 [24.4]	1.3	0.6 – 2.8	0.495	-	-	-
**Group distance (km)**	<10.0	34/148 [23.0]	1.0					
	≥ 10.0	14/84 [16.7]	0.7	0.3 – 1.3	0.249	-	-	-
**Starting 1st line regimen**	Zidovudine based	29/118 [24.6]	1.0					
	Stavudine based	19/114 [16.7]	0.6	0.3 – 1.2	0.136	-	-	-
***Baseline* WHO staging**	I/II	24/131 [18.3]	1.0					
	III/IV	24/100 [24.0]	1.4	0.7 – 2.7	0.293	-	-	-
***Baseline* BMI groups (Kg/m^2)**	< 18.5	22/95 [23.2]	1.0					
	≥ 18.5	26/136 [19.1]	0.8	0.4 – 1.5	0.458	-	-	-
***Baseline* CD4 groups (cells/uL)**	< 100	22/96 [22.9]	1.0					
	≥ 100	26/134 [19.4]	0.8	0.4 – 1.5	0.519	-	-	-
**Duration on ART (months)**	<12.0	21/81 [25.9]	1.0					
	≥ 12.0	27/151 [17.9]	0.6	0.3 – 1.2	0.154	-	-	-
**Drug substitution**	No	33/168 [19.6]	1.0					
	Yes	15/64 [23.4]	1.3	0.6 – 2.5	0.524	-	-	-
***Follow up* WHO staging**	Stage I/II	22/132 [16.7]	1.0					
	Stage III/IV	26/100 [26.0]	1.7	0.9 – 3.3	0.084	-	-	-
***Follow up* BMI groups (Kg/m^2)**	< 18.5	10/36 [27.8]	1.0					
	≥ 18.5	38/196 [19.4]	0.6	0.3 – 1.4	0.267	-	-	-
***Follow up* CD4 groups (cells/uL)**	< 350	31/131 [23.7]	1.0					
	≥ 350	8/70 [11.4]	0.4	0.2 – 1.0	0.031	-	-	-
**MPR adherence**	≥ 95% (Satisfactory)	28/181 [15.5]	1.0			1.0		
	<95% (Unsatisfactory)	15/43 [34.9]	2.9	1.4 – 6.2	0.006	2.5	1.0 – 6.2	0.050

***Baseline* refers to indicators at ART initiation; *Follow up* refers to indicators at the time of sampling; *Likelihood Ratio Test p-value; BMI (Body Mass Index); WHO (World Health Organization); MPR (Medicine Possession Ratio). Missing data; Baseline WHO staging (n=1 [0.4%]), Baseline BMI (n= 1 [0.4%]), Baseline CD4 count (n=2 [0.9%]), CD4 count (n=31 [13.4%]) and MPR adherence (n=8 [3.5%]).**

Supplementary table 2: Distribution and correlates of HIV-1 acquired drug resistance (if genotyping was only done for samples with viral load ≥1000 copies/ml) among first-line antiretroviral experienced adults at a rural HIV clinic in coastal Kenya (N=230).

		Acquired drug resistance [row %]		
**Characteristic**	**Categories**	**No (n=202)**	**Yes (n=28)**	***P-value**
**Gender**	Male	51 [94.4]	3 [5.6]	
	Female	151 [85.8]	25 [14.2]	0.100
**Age group (years)**	15.0 – 34.9	61 [76.3]	19 [23.7]	
	≥ 35.0	141 [94.0]	9 [6.0]	<0.001
**Marital status**	Single	13 [68.4]	6 [31.6]	
	Married (monogamous/polygamous)	114 [87.0]	17 [13.0]	
	Separated/Divorced/Widowed	75 [93.8]	5 [6.3]	0.013
**Religion**	Christian	132 [87.4]	19 [12.6]	
	Muslim	36 [87.8]	5 [12.2]	
	Others	34 [89.5]	4 [10.5]	1.000
**Education status**	Primary schooling/Less	163 [88.1]	22 [11.9]	
	Secondary/Higher	39 [86.7]	6 [13.3]	0.801
**Group distance (km)**	<10.0	129 [87.2]	19 [12.8]	
	≥10.0	73 [89.0]	9 [11.0]	0.834
**Starting 1st line regimen**	Zidovudine based	102 [86.4]	16 [13.6]	
	Stavudine based	100 [89.3]	12 [10.7]	0.550
***Baseline* WHO staging**	I/II	117 [90.0]	13 [10.0]	
	III/IV	84 [84.9]	15 [15.2]	0.309
***Baseline* BMI groups (Kg/m^2)**	< 18.5	83 [89.3]	10 [10.8]	
	≥ 18.5	118 [86.8]	18 [13.2]	0.683
***Baseline* CD4 groups (cells/uL)**	<100	82 [86.3]	13 [13.7]	
	> 100	118 [88.7]	15 [11.3]	0.683
**Duration on ART (months)**	< 12.0	70 [86.4]	11 [13.6]	
	≥ 12.0	132 [88.6]	17 [11.4]	0.675
**Drug substitution**	No	145 [87.4]	21 [12.6]	
	Yes	57 [89.1]	7 [10.9]	0.825
***Follow up* WHO staging**	Stage I/II	119 [91.5]	11 [8.5]	
	Stage III/IV	83 [83.0]	17 [17.0]	0.066
***Follow up* BMI groups (Kg/m^2)**	< 18.5	30 [83.3]	6 [16.7]	
	≥ 18.5	172 [88.7]	22 [11.3]	0.404
***Follow up* CD4 groups (cells/uL)**	< 350	111 [85.4]	19 [14.6	
	≥ 350	64 [91.4]	6 [8.6]	0.267
**MPR adherence**	≥ 95% (Satisfactory)	162 [90.5]	17 [9.5]	
	< 95% (Unsatisfactory)	32 [74.4]	11 [25.6]	0.009
**Viral load (log 10, copies/ml)**	0.00 – 4.00	190 [96.0]	8 [4.0]	
	> 4.00	12 [37.5]	20 [62.5]	<0.001

***Baseline* refers to indicators at ART initiation; *Follow up* refers to indicators at the time of sampling; *Fisher's exact p-value; BMI (Body Mass Index); WHO (World Health Organization) MPR (Medicine Possession Ratio). Missing data; Baseline WHO staging (n=1 [0.4%]), Baseline BMI (n= 1 [0.4%]), Baseline CD4 count (n=2 [0.9%]), CD4 count (n=31 [13.4%]) and MPR adherence (n=8 [3.5%]).**
